# Rheumatic heart disease anno 2020: Impacts of gender and migration on epidemiology and management

**DOI:** 10.1111/eci.13374

**Published:** 2020-08-29

**Authors:** Reuben K. Mutagaywa, Anna‐Maria Wind, Apolinary Kamuhabwa, Maarten J. Cramer, Pilly Chillo, Steven Chamuleau

**Affiliations:** ^1^ School of Medicine Muhimbili University of Health and Allied Sciences Dar Es Salaam Tanzania; ^2^ Division of Heart and Lung Department of Cardiology Faculty of Medicine University Medical Centre Utrecht Utrecht The Netherlands; ^3^ School of Pharmacy Muhimbili University of Health and Allied Sciences Dar Es Salaam Tanzania; ^4^ Division of Heart and Lung Department of Cardiology Faculty of Medicine Amsterdam University Medical Centre Amsterdam The Netherlands

**Keywords:** gender, impact, migration, review, rheumatic mitral stenosis, sex

## Abstract

**Background:**

The epidemiology and management of diseases can be influenced by social demographic factors. Gender and migration are among these factors.

**Methods:**

We aimed at reviewing the impacts of gender and migration on rheumatic heart disease (RHD) epidemiology and management by a nonsystematic literature review of published studies on RHD worldwide. Our PubMed search terms included RHD pathophysiology, diagnosis, complications, management or prevention, combined with words ‘rheumatic mitral stenosis (MS)’, ‘outcomes after percutaneous balloon mitral valvuloplasty (PBMV)’, ‘gender or sex difference’ and ‘migration’. The reporting of this study conforms to SANRA (the Scale for Assessment of Narrative Review Articles) guidelines.

**Results:**

We retrieved eight studies about the impact of sex on outcomes after PBMV. All of these studies showed a female predominance for RHD. Two studies showed that there is no impact, three studies showed female sex as a predictor of poor outcomes, and the other three showed male sex a predictor of poor outcomes. Although RHD is reported to be eradicated in the developed countries, 2.1% of refugees recently screened for RHD in Italy were found to have subclinical RHD. This prevalence is similar to those found in India (2.0%), Cambodia (2.2%) and Mozambique (3%).

**Conclusions:**

There are contradicting results for outcomes after PBMV between males and females. It is not clear whether sex difference plays a role in pathophysiology, diagnosis, management and prognosis of MS. Migration has impacts on epidemiology and management of RHD. Further studies are required in these two fields to explore their relationship to RHD.

## 1 INTRODUCTION

Rheumatic heart disease (RHD) is an important cause of cardiac morbidity and mortality among children and young adults. Worldwide, RHD affects 15.6 million people yearly, and it is responsible for about 300 000 deaths each year.^1^, ^2^ RHD has been eradicated in developed countries mainly attributed to improved living standards and widespread use of antibiotics.^3^ However, recent studies have shown that globalization, migration and refugee crises have led to evolving of RHD in developed countries making RHD a global health problem.^4^, ^5^ Antonio et al^6^ reported that cardiovascular diseases (CVDs) are major health problems among immigrants in developed nations with RHD being a major reason for hospitalization for cardiac operation. Moreover, RHD has been reported in middle‐class children in Utah, United States of America,^7^, ^8^, ^9^ and outbreaks have been recently reported in Trieste, Italy.^10^ More recently, in a study done by Condemi et al^11^ in Italy, it was found that the prevalence of RHD was similar to that found in resource‐limited countries. Indeed, Marijon^12^ warned that ‘the affluent world cannot afford complacency; large population movements and refugee crises can displace persons with RHD to developed nations’. RHD is not confined to low income or tropical nations, but it should raise concern in middle‐ and high‐income countries.

Rheumatic heart disease is still prevalent in many parts of developing countries, including sub‐Saharan Africa where the prevalence is 1‐3 for every 1000 school children.^2^ In these countries, RHD is seen in people with poor socioeconomic status, living in overcrowded conditions, with limited access to health care.^13^, ^14^ Patients present late to tertiary facilities when they have indications for invasive interventions due to the distal sequelae of RHD.^13^ For situations in which open heart surgery (OHS) cannot be performed, procedures like percutaneous balloon mitral valvuloplasty (PBMV) can be performed for MS.

The pathogenesis of acute rheumatic fever (ARF) and its sequelae RHD remains incompletely understood. Evidence supports the view that ARF is the result of an autoimmune response to pharyngeal infection with group A Streptococcal (GAS) in a genetically susceptible individual, which is mediated through molecular mimicry.^15^ Poverty and social disadvantages are among the strongest predisposing factors for developing ARF.^16^ Patients with ARF present with a combination of fever, joints pain, carditis, chorea and skin manifestations (the Jones criteria).^17^ Carditis commonly involves the mitral (35%) and aortic valves (12%) while tricuspid and pulmonary valves are rarely involved.^18^ In regions with a high prevalence of RHD, it is common to see patients in whom more than one valve is affected. Complications of RHD include atrial fibrillation, stroke, heart failure, pulmonary hypertension and infective endocarditis.^13^ Treatment of ARF includes treatment of GAS infection^19^ and arthritis as well as preventing recurrent attacks of ARF with a monthly injection of Benzathine penicillin G.^20^ Therapeutic strategy in RHD involves the management of its associated complications by medications and/or OHS or PBMV (for MS) as per available guidelines.^21^, ^22^

Percutaneous balloon mitral valvuloplasty is a safe and effective management for rheumatic MS. It has excellent procedural success rate (defined by the composite endpoint of a final mitral valve area ≥1.5 cm^2^ without mitral regurgitation [MR] of more than grade 2) of 90% to 95%^23^, ^24^, ^25^ with good immediate^26^ and long‐term outcomes^21^, ^22^; event‐free survival rate of 90% at 5‐7 years,^27^ 61% to 96% at 10 years^28^, ^29^, ^30^ and 43% to 79% at 15 years.^25^, ^28^ For this reason, PBMV is considered standard of care for selected patients with rheumatic MS with Wilkins score <8 and mild MR.^21^, ^22^ Wilkins score^31^ is an echocardiographic method used to predict the success of PBMV in the setting of rheumatic MS. The score has four components: leaflet mobility, thickness, calcification and sub‐valvular thickening each of which is graded from 1 to 4 with a total score being the sum of the items and ranging from 4 to 16. The determinants of procedural success are multifactorial depending on correct patient selection and some anatomical and clinical criteria.^32^, ^33^ The influence of sex on diagnosis, management, the progression of MS and on short‐ and long‐term post‐PBMV results remain unclear.^34^, ^35^ While a study by Palacios et al^32^ reported sex as an independent predictor of PBMV success, other studies^33^, ^36^ did not confirm that observation. Indeed, the limited data available in the literature are inconsistent. Moreover, the recent European Society of Cardiology and the American Heart Association guidelines in the management of valvular heart disease did not mention sex as a predictor of outcomes post interventions.^21^, ^22^, ^37^, ^38^

This article aimed at reviewing the impacts of gender and migration on rheumatic heart disease (RHD) epidemiology and management, with emphasis on rheumatic MS by a nonsystematic literature review of PubMed published studies on RHD worldwide. Additional searches were done in Google Scholar for categories that yielded few results in PubMed. This review article adhered to SANRA guidelines, a brief critical appraisal for the assessment of nonsystematic articles.^39^

## 2 SEX DIFFERENCES IN RHEUMATIC HEART DISEASE: FOCUSING ON MITRAL STENOSIS

### 2.1 Epidemiology and risk factors

Sex differences are frequently encountered in RHD as shown by the female predominance in a ratio of 2:1 in studies concerning PBMV.^32^, ^35^, ^40^, ^41^ There is a difference in the epidemiological presentation of MS according to regions. In Africa, the prevalence of MS is up to 26% among children with RHD aged 6 to 10 years and shows female predominance with an early presentation in life (mean age of 28 years in male, 31 years in female) but with already advanced disease mostly in New York Heart Association (NYHA) functional class II‐III.^13^, ^14^ For South Asia, MS also shows female predominance and clinical presentation similar to Africa but with a higher mean age of 39 years, similar to that seen in western countries.^42^ According to the Euro Heart Survey, the prevalence of MS in the Mediterranean and Eastern European countries is high, accounting for 12% of valvular diseases in Europe.^43^ In these countries, MS exhibits female predominance but the age at presentation is late, with only 4.7% presenting before 40 years.^44^ These patients had low mean NYHA class (1.5) but they deteriorate with age and with the presence of comorbidities. The incidence of atrial fibrillation (AF) is higher (72%) than that seen among patients from Africa (28%)^13^, ^14^ and Asia (32%).^42^ Thromboembolic events occur in 3.2%^45^ and 12.3%^42^ of patients in Africa and Asia, respectively.

### 2.2 Mitral valve anatomy and its effects on management

Although the pathophysiology of rheumatic MS has long been known, its clinical presentation is known to vary significantly by sex. In females, isolated MS is more common, while mixed mitral valve (ie MS and mitral regurgitation) is common in males.^35^, ^43^ In a study done by Cruz‐Gonzalez et al,^46^ females had good anatomy for PBMV based on Wilkins score but short‐term post‐PBMV outcomes were worse among females compared to males (success rate of 69% vs. 83%). They speculated that this may be related to an increased incidence of post‐PBMV MR among females (9.4% vs. 4%) that in turn may reflect the slightly higher effective balloon dilatation to body surface ratio in females. Moreover, it has been reported that morphology and severity of mitral valve (MV) prolapse vary by sex.^47^ It can be postulated that morphology and outcomes of MS also vary by sex.^46^ However, the mechanisms of these differences need to be explored.

### 2.3 Disease presentation, diagnosis and management

Mitral stenosis presentation differs between different geographical settings. In Africa and Asia, patients present with advanced stages of heart failure (HF) with paroxysmal nocturnal dyspnoea and pulmonary oedema being present in 30.5% and 16.7% of patients, respectively. In developed countries, patients present mainly in a lower class of HF, NYHA class II.

Recently, gender medicine has attracted attention in the way CVDs are managed, as several studies^47^, ^48^, ^49^, ^50^ have shown differences between males and females in terms of disease presentation, response to treatment and prognosis. However, it remains unclear whether this gender difference is a true biological sex difference or due to sex bias in medical care, in which females are less likely to get correct screening and prevention strategies, early diagnosis, and evidence‐based medicine like for instance indications for PBMV for rheumatic MS.

According to Kislitsina et al,^48^ a significant sex difference remains in MV pathology in which females have more RHD than males. When compared to males, females are older, have more symptoms, have more comorbidities, lately referred for intervention and therefore presenting with advanced disease. The reasons for late referral could be that females are well known to have a different presentation (higher prevalence of preserved left ventricular function^49^, ^51^) and less clear symptoms (women with cardiac failure are likely to report symptoms of dyspnoea more vaguely^50^) and have poor socioeconomic status.

A study of 3761 patients by Seeburger et al^52^ revealed that females had more MS and MV leaflet calcification than males. The authors postulated that higher MV calcification in females could be explained by differences in calcium metabolism and bone resorption among postmenopausal females.

The diagnostic approach for rheumatic MS is similar in males and females. An exception is in pregnant women in which an expert professional should be involved to make a risk prediction, if possible before pregnancy because pregnancy increases risk of HF and death.^53^

**Figure 1:**
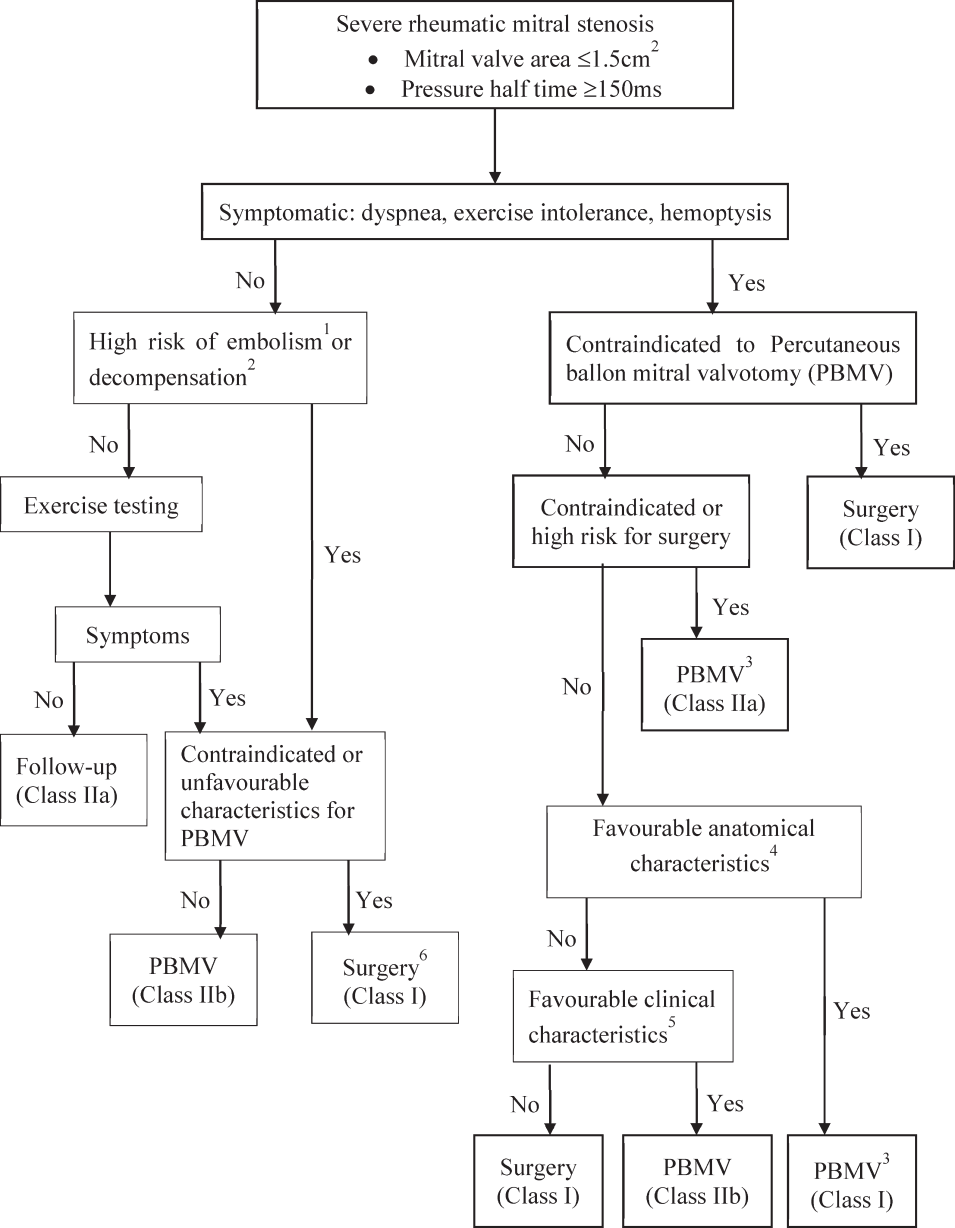
Management of clinically significant mitral stenosis. ^1^Thromboembolism: past embolism, atrial fibrillation, dense echo in left atrium; ^2^decompensation: pulmonary hypertension > 50 mmHg, major noncardiac operation, intention for pregnancy; ^3^Consider commissurotomy by experienced surgeons or in patients contraindicated for PBMV; ^4^Mitral valve orifice <1.5 cm^2^, no left atrium clots, MR < grade 2, mild calcification, no fused commissures; ^5^PBMV for symptomatic patients contraindicated to operation, mitral valve operation for symptomatic patients unsuitable for PBMV; ^6^Surgery if symptomatic at less physical activity and low surgical risk. For the strength of recommendation: class I means the procedure should be performed, class IIa means it is reasonable to perform the procedure, and class IIb means the procedure may be considered (Redrawn from: Baumgartner et al^21^ and Nishimura et al^22^ 161 × 207mm (300 × 300 DPI)

Management of clinically significant MS is as shown in Figure 1.^21^, ^22^ As the figure shows, the only sex difference to take into consideration during management is the presence of pregnancy.

### 2.4 Imaging considerations

There are no sex‐specific differences in the aspect of imaging in a patient with rheumatic MS. Management of patients with rheumatic MS requires the use of echocardiography and multimodality imaging to ensure a thorough assessment of the MV morphology and MS severity.^54^ Usually, 2‐dimension echocardiography (2DE) by transthoracic (TTE) or transesophageal (TEE) is the imaging of choice. Due to its limitations like poor acoustic window and the effects of haemodynamic conditions on Doppler studies, 3‐Dimension echocardiography (3DE) is implicated. TEE is useful for the assessment of left atrial appendage for thrombi and for guiding interventions like PBMV. Multidetector computed tomography (MDCT) can be used as an alternative or complementary method in patients with poor acoustic windows. Cardiovascular magnetic resonance (CMR) is the third choice if echo and MDCT are inconclusive.^54^ However, particular attention should be paid to pregnant women in whom echocardiography and CMR appear to be safe and are not associated with adverse foetal effects owing to lack of radiations.^53^

### 2.5 Approach to interventional management

#### 2.5.1 Percutaneous balloon mitral valvuloplasty

Historically, chronic RHD was being treated with surgical interventions. The balloon for MS was introduced in the early 1980s, became the treatment of choice and offered superior results over open commissurotomy in patients with severe MS and conditions suitable for valvotomy.^55^ PBMV is important in developing countries where haemodynamically severe MS presents earlier in life, and young patients have thickened valve leaflets presenting with or without concurrent regurgitation.^56^ It is also a bridging therapy to OHS of MS patients during pregnancy, postponement of valvular replacement for females to finish their childbearing time (in avoidance of anticoagulation), or in patients who cannot tolerate OHS.^33^, ^55^ Other advantages are those related to its lower cost, lower morbidity and lower procedure‐related mortality.^40^

#### 2.5.2 Surgery

The higher rate of mitral calcification among females lead to many of them to undergo MV replacement than males.^52^ Also, a study done by Cruz‐Gonzalez^46^ showed that females have lower post‐PBMV mitral valve area (MVA) and a higher incidence of post‐PBMV mitral regurgitation the factors which predispose them to MV replacement.

### 2.6 The impacts of gender on immediate and/or long‐term outcomes post‐PBMV for rheumatic MS

Several studies (Table 1) have been conducted to determine the impacts of sex on outcomes after PBMV.

**Table 1:** Studies reporting an association between sex and outcomes after PBMV

Author	Year	Study design	PBMV measure (follow‐up)	Outcome
Cohen et al^40^	1992	Prospective observational study of 147 patients (77% female) who underwent PBMV	Predictors of long‐term event‐free survival (patients without MVR, repeat PBMV or Death) (36 ± 20 mo)	Sex, cardiac rhythm, previous commissurotomy, baseline PAP/LAP, and MPG were not significant multivariate predictors of long‐term outcome
Hernandez et al^35^	1999	Longitudinal observational study of 561 patients (82% female) who underwent PBMV	Long‐term clinical and echocardiographic follow‐up (39 ± 23 mo)	Sex, age, cardiac rhythm, MVA pre‐PBMV were not significant multivariate predictors of long‐term outcome
Yetkin et al^34^	2001	Longitudinal observational study of 156 patients (78% female) who underwent PBMV	Comparison of pre‐ and post‐PBMV characteristics and outcome of male and female patients, 38 mo follow‐up	Restenosis rates were significantly higher among male than female
Palaciao et al^32^	2002	Prospective observational study of 765 patients (81.5% female) who underwent PBMV	Immediate and long‐term clinical follow‐up (4.2 ± 3.7 y)	Male sex, young age, pre‐ PBMV MVA, less degree of pre‐PBMV MR, absence of prior‐commissurotomy, Wilkins score ≤8 were independent predictors of immediate PBMV success
Ignacio Cruz‐Gonzalez et al^59^	2009	Prospective observational study of 1085 patients who underwent PBMV	Immediate and long‐term clinical follow‐up median 3.097 y (interquartile range 1.01‐5.65 y)	Predictors of PBMV success were age <55 y, male sex, NYHA classes I and II, pre‐PBMV MVA ≥1 cm^2^, pre‐PBMV MR grade <2, and Wilkins score ≤8
Ignacio Cruz‐Gonzalez et al^46^	2011	Prospective observational study of 1015 patients (83% female) who underwent PBMV	Immediate and long‐term clinical follow‐up (median 3.1 y)	When compared to men and despite lower Wilkins scores, women who underwent PBMV had lower procedural success.
Bouleti et al^57^	2012	Prospective observational study of 848 patients (85% female) who underwent PBMV	Immediate and long‐term clinical follow‐up median 10.7 y (interquartile range 4.6‐15.8 y)	Predictors of poor late (after good immediate) results post‐PBMV were (among other factors) interaction between male sex and valve calcification.
Fabrizio et al^58^	2014	Prospective observational study of 439 patients (83.3% female) who underwent PBMV	Immediate and long‐term clinical follow‐up (11.7 ± 4.9 y)	Predictors of primary end point (the 20 y incidence of MACE) were: male gender, atrial fibrillation, Wilkins >8, and post‐PBMV MVA <1.75 cm^2^

Abbreviations: LAP, left atrium pressure; MACE, major adverse cardiac events; MR, mitral regurgitation; MVA, mitral valve area; MVR, mitral valve replacement; NYHA, New York Heat Association; PAP, pulmonary artery pressure; PBMV, percutaneous balloon mitral valvuloplast.

#### 2.6.1 Studies showing that sex is not a predictor of outcomes after PBMV

Older studies^35^, ^40^ reported that sex is not a predictor of long‐term outcomes after PBMV. Hernandez et al^35^ did a univariate analysis of several variables (age, sex, cardiac rhythm, previous commissurotomy, Wilkins score, balloon size, and post‐procedural mitral valve area and MR) as predictors of event‐free survival and found that there was no significant difference between male and female (relative risk for female 1.22, 95% CI 0.67‐2.22, *P* = .1213) on outcomes after PBMV. Only Wilkins score was an independent predictor of outcomes on multivariate analysis.

#### 2.6.2 Studies showing that male sex is a predictor of poor outcomes after PBMV

Yetkin et al^34^ found that the restenosis rates in males were higher than in females at 20% and 9%, respectively (*P* < .05). Males were older, had high Wilkins and low pre‐PBMV MVA. Generally, males had unfavourable pre‐procedural clinical and anatomical characteristics except for pre‐PBMV pulmonary arterial pressure (PAP). Authors argued on their findings of the effect of male sex on outcomes that could be due to smaller sample, the study was limited to patients who underwent successful PBMV, and evaluation of different outcome measures.

Bouleti et al^57^ found that 20 years after successful PBMV, 30% of patients still had good functional results (survival without cardiovascular death, MV surgery and repeat PBMV). Interaction between male sex and valve calcification was found to be among the predictors of poor late functional results (adjusted hazard ratio (HR) 2.3, 95% CI 0.6‐3.2, *P* < .0001) showing that the impact of valve anatomy is stronger in men. Other predictors were higher final trans‐mitral mean pressure gradient, the interaction between age and final MVA, interaction between AF and NYHA class.

In a study done by Fabrizio Tomai et al,^58^ among 439 patients for a mean follow‐up of 11 years after successful PBMV with primary endpoints of major adverse cardiovascular events (MACE) including cardiovascular death and need for MV surgery or repeat PBMV; predictors of the primary endpoint were as follows: male sex (HR 1.65, 95% CI 1.17‐2.32, *P* = .004), AF, Wilkins >8 and post‐PBMV MVA <1.75 cm^2^.

#### 2.6.3 Studies showing that female sex is a predictor of poor outcomes after PBMV

Palacio et al^32^ showed that when compared to females, male sex was among the predictors of immediate PBMV success (71.7% for the overall group) both on univariate analysis (*P* = .002) and on multivariate analysis (odds ratio 1.92 and 95% confidence interval 1.19‐3.13, *P* = .008). However, male sex was not among the independent predictors of long‐term mortality. Male comprised 19.5% (n = 182) of the study population; therefore, results could be affected by their smaller number.

Ignacio Cruz‐Gonzalez et al^59^ used a multifactorial score to predict success and long‐term outcomes of PBMV. Independent predictors of poor PBMV outcomes were age >55 years, female sex, NYHA classes ≥III, pre‐PBMV MVA <1 cm^2^, pre‐PBMV MR grade >2 and Wilkins ≥8.

Ignacio et al^46^ showed that when compared to men and despite lower Wilkins scores, women who underwent PBMV had lower procedural success (69% Vs 83%, OR 0.04, 95% CI 0.19‐0.74, *P* = .02), achieved a smaller post‐procedural MVA (73.7% Vs 86.9%, OR 0.14, 95% CI 0.23‐0.72, *P* = .002), and had higher incidence of post‐procedural MR (9.4% Vs 4%,OR 2.41, 95% CI 1.0‐5.83, *P* = .05). However, the difference between the two sexes was not observed for long‐term outcomes (mortality, need for repeat PBMV or composite adverse outcome endpoint of mortality). Male comprised 17% (n = 176) of the study population; therefore, results could be affected by their smaller proportion.

## 3 MIGRATION AND ITS EFFECTS ON DISEASE EPIDEMIOLOGY

### 3.1 Effects of migration on the epidemiology of CVD

It is estimated that 68.5 million people worldwide have been driven from their homes due to either conflict, war, persecution or as asylum seekers^60^ It is known that globalization, urbanization and migration affect the epidemiology of CVDs. ^61^ Therefore, it is important to elucidate how they influence the burden and distribution of CVDs and to modify health systems and health service delivery to changing needs. In their review, Anna et al^62^ argued that there are three factors influencing CVD burden in migrant populations: conditions in the country of origin, the migration process, legal status in the host country.

Migration requires new strategies to be implemented to health policy and practice, informed by evidence derived from relevant research to support them. Anna et al^62^ proposed an approach that includes components which should be introduced in welfare and health systems to bring an effective response to the challenges of migration, specifically the changing pattern of CVD. Since immigrants exhibit variations,^63^ CVD care services should be designed and implemented in ways that respond to certain requirements of immigrants, the variations should also be considered when assessing CVD risk profiles and when designing health services which should be culturally and religiously appropriate, accessible^63^ and acceptable. Health services for migrants should be medically and culturally appropriate and should be integrated into the hosting national health system.^64^ There is a need for enhanced cross‐border surveillance, clinical data sharing and the ability to share cross‐country experience and best practices. The authors named these components as ‘responding‐integrating and sharing’ formula.^62^ They proposed, for them to be in use they should be supported by international commitment, training and education of workforces, information and technology, and by research for filling evidence gap.^62^

### 3.2 Effects of migration on epidemiology and management of RHD

About a third of the 68.5 million people who have been forced from their homes worldwide are refugees, over half of them being under the age of 18 years, putting them at high risk of acute rheumatic fever (ARF).^65^

The exact prevalence of RHD among migrant and refugee populations worldwide is difficult to ascertain; however, it is believed to be high. A recent review on the impact of migration on CVD focused on ischaemic heart disease, its risk factors and consequences^62^ but also appreciated on the high burden of RHD in many parts of the world.^13^

Gianfranco De Maio et al^66^ when they screened refugees aged 10‐25 years for RHD in Rome, Italy, they found that 1.7% of these refugees had definitive RHD. The prevalence of subclinical RHD was 2.1% similar to that found in other parts of the world^13^ such as India (2.0%), Cambodia (2.2%) and Mozambique (3%). Similarly, Condemi et al^11^ have recently screened refugees aged 13‐26 years and found a prevalence of ‘definitive RHD’ of 2.6% while that of ‘borderline RHD’ was 18.6%. The burden of borderline cases was markedly higher than that reported elsewhere. It is being reported that of the more than 1 million asylum seekers who moved to Europe in 2015, approximately 1% of them could have RHD.^67^

Since refugee camps and informal settlements are overcrowded, there is a great likelihood for the streptococcus infections which cause ARF and subsequent RHD to spread more easily.^67^ When refugees settle in places where RHD is uncommon or under‐recognized, this may lead to late diagnosis and eventually poor care associated with disease complications.

The effect of migration on RHD goes beyond disease distribution because it also affects disease management. Antibiotic injections to prevent recurrent ARF and sequela of valve damage must be given monthly, necessitating a stable and supportive health service, something which is unlikely among the migrant populations.^67^ The impact of disorganized health services on disease prevalence in vulnerable communities has been reported before: during the breakdown of the Union of Soviet Socialist Republics, RHD prevalence increased tremendously in association with social and economic disruption.^68^ Besides, migrants have a high burden of noncommunicable diseases which may contribute to other heart diseases in the elderly.

## 4 CONCLUSION AND FUTURE PERSPECTIVES

This review showed a female predominance for RHD and contradicting results for outcome after PBMV among the two sexes. It is not clear whether sex difference plays a role in diagnosis, management and prognosis of MS: (a) Is it a true sex biological difference or a sex bias in medical care, in which female are less likely to get correct screening and prevention strategies due to socioeconomic status? (b) Is there a difference in mitral valve calcification between sexes. Further studies need to be done to explore the mechanisms for the differences.

Migration has impacts on RHD: (a) among immigrants in developed nations, RHD is a major reason of admission and cardiac surgery, (b) migration requires new strategies to be implemented to health policy and practice, evidenced by relevant research, (c) a call for developed nations to revive their medical education programs to include RHD because clinicians and health systems have become unfamiliar with the condition. Further studies are required to explore more on the impacts of migration on RHD.

## CONFLICT OF INTEREST

The authors declare no conflict of interest.
